# Evaluating Humoral Immunity against SARS-CoV-2: Validation of a Plaque-Reduction Neutralization Test and a Multilaboratory Comparison of Conventional and Surrogate Neutralization Assays

**DOI:** 10.1128/Spectrum.00886-21

**Published:** 2021-11-17

**Authors:** Emelissa J. Valcourt, Kathy Manguiat, Alyssia Robinson, Yi-Chan Lin, Kento T. Abe, Samira Mubareka, Altynay Shigayeva, Zoë Zhong, Roxie C. Girardin, Alan DuPuis, Anne Payne, Kathleen McDonough, Zhen Wang, Romain Gasser, Annemarie Laumaea, Mehdi Benlarbi, Jonathan Richard, Jérémie Prévost, Sai Priya Anand, Kristina Dimitrova, Clark Phillipson, David H. Evans, Allison McGeer, Anne-Claude Gingras, Chen Liang, Martin Petric, Inna Sekirov, Muhammad Morshed, Andrés Finzi, Michael Drebot, Heidi Wood

**Affiliations:** a Zoonotic Diseases and Special Pathogens, National Microbiology Laboratory, Public Health Agency of Canada, Winnipeg, Manitoba, Canada; b Max Rady College of Medicine, University of Manitoba, Winnipeg, Manitoba, Canada; c Department of Medical Microbiology and Immunology, University of Alberta, Edmonton, Alberta, Canada; d Department of Microbiology, Mount Sinai Hospital, Sinai Health System, Toronto, Ontario, Canada; e Department of Molecular Genetics, University of Toronto, Toronto, Ontario, Canada; f Department of Laboratory Medicine and Molecular Diagnostics, Division of Microbiology, Sunnybrook Health Sciences Centre, Toronto, Ontario, Canada; g Institute of Health Policy, Management and Evaluation, University of Toronto, Toronto, Ontario, Canada; h Faculty of Medicine, University of Toronto, Toronto, Ontario, Canada; i Wadsworth Center, New York State Department of Health, Albany, New York, USA; j Department of Medicine, McGill University, Montreal, Quebec, Canada; k British Columbia Centre for Disease Control Public Health Laboratory, Vancouver, British Columbia, Canada; l Département de Microbiologie, Infectiologie et Immunologie, Université de Montréal, Montreal, Quebec, Canada; m Centre de Recherche du CHUM, Montreal, Quebec, Canada; n Department of Medical Microbiology, University of Manitoba, Winnipeg, Manitoba, Canada; University of Georgia

**Keywords:** COVID-19, immunity, SARS-CoV-2, immunoserology, neutralizing antibodies

## Abstract

The evaluation of humoral protective immunity against SARS-CoV-2 remains crucial in understanding both natural immunity and protective immunity conferred by the several vaccines implemented in the fight against COVID-19. The reference standard for the quantification of antibodies capable of neutralizing SARS-CoV-2 is the plaque-reduction neutralization test (PRNT). However, given that it is a laboratory-developed assay, validation is crucial in order to ensure sufficient specificity and intra- and interassay precision. In addition, a multitude of other serological assays have been developed, including enzyme-linked immunosorbent assay (ELISA), flow cytometry-based assays, luciferase-based lentiviral pseudotype assays, and commercially available human ACE2 receptor-blocking antibody tests, which offer practical advantages in the evaluation of the protective humoral response against SARS-CoV-2. In this study, we validated a SARS-CoV-2 PRNT to assess both 50% and 90% neutralization of SARS-CoV-2 according to guidelines outlined by the World Health Organization. Upon validation, the reference-standard PRNT demonstrated excellent specificity and both intra- and interassay precision. Using the validated assay as a reference standard, we characterized the neutralizing antibody response in specimens from patients with laboratory-confirmed COVID-19. Finally, we conducted a small-scale multilaboratory comparison of alternate SARS-CoV-2 PRNTs and surrogate neutralization tests. These assays demonstrated substantial to perfect interrater agreement with the reference-standard PRNT and offer useful alternatives to assess humoral immunity against SARS-CoV-2.

**IMPORTANCE** SARS-CoV-2, the causal agent of COVID-19, has infected over 246 million people and led to over 5 million deaths as of October 2021. With the approval of several efficacious COVID-19 vaccines, methods to evaluate protective immune responses will be crucial for the understanding of long-term immunity in the rapidly growing vaccinated population. The PRNT, which quantifies SARS-CoV-2-neutralizing antibodies, is used widely as a reference standard to validate new platforms but has not undergone substantial validation to ensure excellent inter- and intraassay precision and specificity. Our work is significant, as it describes the thorough validation of a PRNT, which we then used as a reference standard for the comparison of several alternative serological methods to measure SARS-CoV-2-neutralizing antibodies. These assays demonstrated excellent agreement with the reference-standard PRNT and include high-throughput platforms, which can greatly enhance capacity to assess both natural and vaccine-induced protective immunity against SARS-CoV-2.

## INTRODUCTION

Since its emergence in December 2019, the coronavirus disease 2019 (COVID-19) pandemic has caused millions of cases and fatalities worldwide (https://www.who.int/news/item/30-01-2020-statement-on-the-second-meeting-of-the-international-health-regulations-(2005)-emergency-committee-regarding-the-outbreak-of-novel-coronavirus-(2019-ncov); https://www.who.int/publications/m/item/weekly-epidemiological-update-on-covid-19---26-october-2021). Mitigating transmission of severe acute respiratory syndrome coronavirus-2 (SARS-CoV-2) has proven extremely difficult due to presymptomatic viral shedding (1) and the long infectious period of a large portion of asymptomatically infected individuals (2). These factors severely hinder case detection, self-isolation, and quarantine (3). Studies showed that some immune responses, including viral neutralization, decrease within weeks of infection in both symptomatic and asymptomatic individuals (2, 4–7). However, additional immune responses, including Fc-mediated effector functions, T cell immunity responses, and memory B cell responses, remain detectable for at least 5 months postinfection (8–10). With success of several vaccines in clinical trials (11, 12), as well as hundreds of other vaccine candidates in different stages of development (13, 14), the implications of waning immunity on long-term vaccine-mediated protection will require further investigation. In order to do so, a means to quantify humoral protective immunity to SARS-CoV-2 will be indispensable.

Several laboratory-developed (5, 6, 15) and commercially available (16) enzyme-linked immunosorbent assays (ELISAs) have been developed to detect anti-SARS-CoV-2 antibodies raised upon infection. While these platforms provide a high-throughput means of detecting antibodies against SARS-CoV-2, they are unable to measure the immunological function of SARS-CoV-2-specific antibodies. In contrast, the plaque-reduction neutralization test (PRNT) quantifies levels of neutralizing antibodies capable of blocking the interaction that mediates virus entry into susceptible host cells and subsequent virus replication. For SARS-CoV-2, this interaction involves binding of the receptor binding domain (RBD) of the SARS-CoV-2 spike glycoprotein with the angiotensin-converting enzyme 2 (ACE2) (17). Antibodies targeting non-RBD epitopes of SARS-CoV-2 have also demonstrated low to high levels of neutralization activity (18–20). While the conventional PRNT is often used as the reference standard for the evaluation of virus-neutralizing antibodies, this assay is time-consuming and laborious and requires containment level 3 (CL-3) facilities to work with the risk group-3 pathogen. In contrast, laboratory-developed and commercially available surrogate SARS-CoV-2 neutralization tests, including microneutralization and human ACE 2 receptor-blocking antibody tests, have been developed for high-throughput evaluation of functional immunity against SARS-CoV-2 and may offer advantages in large-scale immunity testing.

In this study, we aimed to validate a laboratory-developed SARS-CoV-2 PRNT to assess both 50% neutralization (PRNT-50) and 90% neutralization (PRNT-90) of SARS-CoV-2 according to guidelines outlined by the World Health Organization (https://www.who.int/medicines/areas/quality_safety/quality_assurance/28092018Guideline_Validation_AnalyticalMethodValidation-Appendix4_QAS16-671.pdf) and characterized the neutralizing antibody response in laboratory-confirmed COVID-19 specimens. Using the validated PRNT as a reference standard, along with a panel of characterized serological specimens, we conducted a small-scale multilaboratory comparison of alternate SARS-CoV-2 PRNTs and surrogate neutralization tests to identify other methods to assess the neutralizing antibody response in SARS-CoV-2 infection.

## RESULTS

### Neutralizing antibody responses of the laboratory-confirmed COVID-19 sample subset quantified by SARS-CoV-2 PRNT.

The reference-standard PRNT was used to test a panel of specimens collected from COVID-19 patients confirmed by molecular testing. Of the laboratory-confirmed COVID-19 specimens, 82.1% and 54.1% demonstrated 50% and 90% neutralization of SARS-CoV-2 by PRNT-50 and PRNT-90, respectively (Table 1). The proportions of laboratory-confirmed COVID-19 specimens eliciting 50% neutralization were 66.7%, 75.0%, 95.0%, 93.8%, 85.0%, 86.4%, 100%, and 85.7% for samples collected at 1 to 7, 8 to 14, 15 to 21, 22 to 28, 29 to 35, 36 to 42, 43 to 49, and ≥50 days post-symptom onset, respectively. The proportion of laboratory-confirmed COVID-19 specimens eliciting 90% neutralization were 30.8%, 50.0%, 65.0%, 75.0%, 80.0%, 54.5%, 50.0%, and 61.9% for samples collected at 1 to 7, 8 to 14, 15 to 21, 22 to 28, 29 to 35, 36 to 42, 43 to 49, and ≥50 days post-symptom onset, respectively.

**TABLE 1:** Proportion of molecularly confirmed COVID-19 specimens eliciting 50% and 90% neutralization by PRNT-50 and PRNT-90

PRNT type	Proportion of specimens^*a*^ eliciting neutralization at days post-symptom onset:								
	1–7 (*n* = 39)	8–14 (*n* = 60)	15–21 (*n* = 20)	22–28 (*n* = 16)	29–35 (*n* = 20)	36–42 (*n* = 22)	43–49 (*n* = 20)	≥50 (*n* = 21)	Total (*n* = 218)
PRNT-50	66.7% (26; 49.8–80.9%)	75.0% (45; 62.1–85.3%)	95.0% (19; 75.1–99.9%)	93.8% (15; 69.8–99.8%)	85.0% (17; 62.1–96.8%)	86.4% (19; 65.1–97.1%)	100% (20; 83.2–100%)	85.7% (18; 63.7–97.0%)	82.1% (179; 76.4–87.0%)
PRNT-90	30.8% (12; 17.0–47.6%)	50.0% (30; 36.8–63.2%)	65.0% (13; 40.8–84.6%)	75.0% (12; 47.6–92.7%)	80.0% (16; 40.8–84.6%)	54.5% (12; 32.2–75.6%)	50.0% (10; 25.7–72.8%)	61.9% (13; 38.4–81.9%)	54.1% (118; 47.3–60.9%)

a Number of COVID-19 specimens detected and 95% confidence intervals are indicated in parentheses.

**FIG 1:**
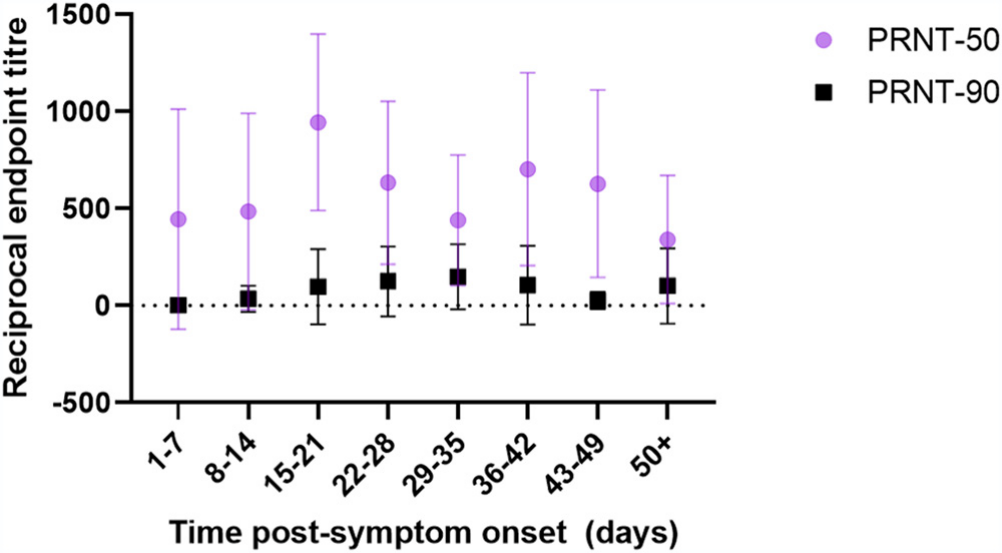
Average neutralizing antibody titers in molecularly confirmed COVID-19 patients capable of 50% (PRNT-50) and 90% (PRNT-90) neutralization of SARS-CoV-2 at different times post-symptom onset. Specimens with a negative titer of <20 were given a reciprocal endpoint titer of 0 for statistical analyses. The dotted line represents the limit of detection.

The average reciprocal endpoint titers capable of neutralizing 50% SARS-CoV-2 (PRNT-50 titer) were 446, 483, 944, 634, 440, 703, 628, and 341 for specimens collected at collected at 1 to 7, 8 to 14, 15 to 21, 22 to 28, 29 to 35, 36 to 42, 43 to 49, and ≥50 days post-symptom onset, respectively (Fig. 1). The average PRNT-50 titers of specimens collected between 15 and 21 days post-symptom onset were significantly higher than those of specimens collected between 1 and 7 (*P* < 0.0001), 8 to 14 (*P* < 0.0001), 29 to 35 (*P* = 0.0008), and ≥50 (*P* < 0.0001) days post-symptom onset, respectively.

The average reciprocal endpoint titers capable of neutralizing 90% SARS-CoV-2 were 3, 35, 98, 125, 149, 106, 26, and 102 for specimens collected at collected at 1 to 7, 8 to 14, 15 to 21, 22 to 28, 29 to 35, 36 to 42, 43 to 49, and ≥50 days post-symptom onset (Fig. 1). Average PRNT-90 titers between specimens collected from laboratory-confirmed COVID-19 at different times post-symptom onset were not significantly different.

### Validation of the reference standard SARS-CoV-2 PRNT.

The sensitivity of the PRNT-50 and PRNT-90 for laboratory-confirmed COVID-19 specimens was 82.1% and 54.1%, respectively (Table 2). Both PRNT-50 and PRNT-90 demonstrated 100% specificity. Within the same assay, the PRNT-50 demonstrated excellent intraassay precision, with 100%, 100%, and 86.1% of results within 4-fold of, within 2-fold of, and equal to the median test result, respectively (Table 3). The PRNT-90 also demonstrated sufficient intraassay precision, with 100%, 98.6%, and 84.7% of results within 4-fold of, within 2-fold of, and equal to the median test result. Both the PRNT-50 and PRNT-90 surpassed the validation cutoff, which required ≥90% of results generated to be within 2-fold of the median test result (https://www.who.int/medicines/areas/quality_safety/quality_assurance/28092018Guideline_Validation_AnalyticalMethodValidation-Appendix4_QAS16-671.pdf). The accuracy of the reference-standard PRNT when using the PRNT-50 analysis was 88.8% (51.8 to 99.7%) and thus was considered to have sufficient accuracy (Table 2). The absolute differences between the log_2_ observed and log_2_ expected values for the first SARS-CoV-2 specimen were 0.36, 0.49, and 0.19, for 1:4, 1:16, and 1:64 dilutions, respectively. The absolute differences between the log_2_ observed and log_2_ expected values for the second SARS-CoV-2 specimen were 1.81, 1.81, and 2.13, for 1:4, 1:16, and 1:64 dilutions, respectively. The absolute differences between the log_2_ observed and log_2_ expected values for the first SARS-CoV-2 specimen were 0.32, 0.00, and 0.00 for 1:4, 1:16, and 1:64 dilutions, respectively. However, accuracy could not be calculated for the PRNT-90 analysis since the expected titers for 1:4, 1:16, and 1:64 were below the limit of detection of the assay (1:20).

**TABLE 2:** Sensitivity, specificity, and accuracy of SARS-CoV-2 PRNT^*a*^

Characteristic	PRNT-50	PRNT-90
Sensitivity^*b*^ (*n* = 218)	82.1% (179; 76.5–87.0%)	54.1% (118; 47.3–60.9%)
Specificity^*c*^ (*n* = 52)	100% (52; 93.1–100%)	100% (52; 93.1–100%)
Accuracy^*d*^ (*n* = 9)	88.8% (8; 51.8–99.7%)	NA

a Due to various host responses, specimens within the molecularly confirmed COVID-19 subset may include both nAb+ and nAb− specimens. NA, not applicable.

b Sensitivity: percentage of molecularly confirmed COVID-19 specimens which tested positive by SARS-CoV-2 PRNT.

c Specificity: specimens collected from healthy patients prior to the COVID-19 pandemic which tested negative by SARS-CoV-2 PRNT.

d Accuracy: 3 specimens positive for SARS-CoV-2 nAbs diluted at 1:4, 1:16, and 1:64 and undiluted and tested 4 times. A result was considered accurate if the absolute difference between the log_2_ expected and log_2_ observed value was ≤2.00. Accuracy of the PRNT-90 could not be assessed since the expected titers of diluted specimens fell below the limit of detection of the assay (1:20). Number of specimens and 95% confidence intervals are indicated in parentheses.

**TABLE 3:** Intraassay and interassay precision of the SARS-CoV-2 PRNT

Precision type	Assessment	PRNT-50	PRNT-90
Intraassay precision (*n* = 24)	Percentage of results within 4-fold of median	100%	100%
	Percentage of results within 2-fold of median (validation cutoff: ≥90%)	100%	98.6%
	Percentage of results equal to median	86.1%	84.7%
Interassay precision (*n* = 18)	Percentage of results within 4-fold of median	98.2%	98.2%
	Percentage of results within 2-fold of median (validation cutoff: ≥90%)	98.2%	90.7%
	Percentage of results equal to median	80.0%	68.5%

Between several assay runs, the PRNT-50 demonstrated sufficient interassay precision, with 98.2%, 98.2%, and 80.0% of results within 4-fold of, within 2-fold of, and equal to the median test result, respectively (Table 3). The PRNT-90 also demonstrated sufficient interassay precision, with 98.2% and 90.7% of results within 4-fold and 2-fold of the median test result; 68.5% of results by PRNT-90 were equal to the median test result. Both the PRNT-50 and PRNT-90 surpassed the validation cutoff, which required ≥90% of results generated to be within 2-fold of the median test result. The reference-standard PRNT demonstrated no reactivity with the COVID-19+/neutralizing antibody-negative (nAb−), SARS-CoV-1, hepatitis, HIV, syphilis, and preoutbreak sera that were used in the panel for the multilaboratory assay comparison.

### Small-scale multilaboratory comparison of conventional and surrogate SARS-CoV-2 neutralization assays.

Alternate PRNT conducted by University of Alberta (U of A) demonstrated 100% congruence (*P* < 0.0001) and perfect interrater agreement with the reference-standard PRNT (κ-value 1.000) as shown in Table 4. This assay detected 100% of laboratory-confirmed COVID-19 nAb+ specimens and did not cross-react with any specimens from the COVID-19 nAb−, SARS-CoV-1, hepatitis, HIV, or syphilis subsets. This assay also did not show any reactivity with sera collected from healthy patients prior to the COVID-19 pandemic. The alternate PRNT conducted by Wadsworth demonstrated 97.5% congruence (*P* < 0.0001) and almost perfect interrater agreement with the reference-standard PRNT (κ-value 0.950). This assay detected 100% of laboratory-confirmed COVID-19 nAb+ specimens and did not show any reactivity with sera other than the laboratory-confirmed COVID-19 nAb− specimen.

**TABLE 4:** Congruence of alternate and surrogate SARS-CoV-2 neutralization platforms with the reference-standard PRNT-50^*a*^

Specimen subset	Congruence^*b*^ of:								
	Alternate PRNT assays		Microneutralization assay	Surrogate virus neutralization tests					ELISA
	PRNT-50 (Wadsworth)	PRNT-50 (U of A)	cVNT (BC CDC)	FACS-based sVNT (CRCHUM)	Luciferase-based lentivirus sVNT (McGill)	Luciferase-based lentivirus sVNT (CRCHUM)	Luciferase-based lentivirus sVNT (Mount Sinai)	Genscript hACE2 receptor-blocking antibody test (McGill)	Anti-RBD IgG ELISA (CRCHUM)
COVID-19 nAb+ (*n* = 19)	100% (19)	100% (19)	94.7% (18)	100% (19)	100% (19)	89.5% (17)	100% (19)	100% (19)	100% (19)
COVID-19 nAb− (*n* = 1)	0% (0)	100% (1)	100% (1)	0% (0)	0% (0)	100% (1)	0% (0)	0% (0)	100% (1)
SARS-CoV-1 (*n* = 2)	100% (2)	100% (2)	100% (2)	0% (0)	50% (1)	100% (2)	100% (2)	50% (1)	50% (1)
Hepatitis (*n* = 5)	100% (5)	100% (5)	80% (4)	100% (5)	100% (5)	100% (5)	100% (5)	100% (5)	100% (5%)
HIV (*n* = 3)	100% (3)	100% (3)	100% (3)	100% (3)	33.3% (1)	100% (3)	100% (3)	100% (3)	100% (3)
Syphilis (*n* = 5)	100% (5)	100% (5)	100% (5)	100% (5)	40% (2)	80% (4)	0% (0)	100% (5)	100% (5)
Preoutbreak sera (*n* = 5)	100% (5)	100% (5)	100% (5)	100% (5)	100% (5)	100% (5)	100% (5)	100% (5)	100% (5)
Total congruence (*n* = 40)	97.5% (39; <0.0001)	100% (40; <0.0001)	95.0% (38; <0.0001)	92.5% (37; <0.0001)	82.5% (33; <0.0001)	92.5% (37; <0.0001)	85.0% (34; <0.0001)	95.0% (38; <0.0001)	97.5% (39; <0.0001)
κ-value	0.950 (0.853–1.000)	1.000 (1.000–1.000)	0.900 (0.764–1.000)	0.851 (0.690–1.000)	0.655 (0.437–0.873)	0.849 (0.685–1.000)	0.704 (0.495–0.912)	0.900 (0.766–1.000)	0.950 (0.853–1.000)
									
Interrater agreement	Almost perfect	Perfect	Almost perfect	Almost perfect	Substantial	Almost perfect	Substantial	Almost perfect	Almost perfect

a COVID-19 nAb+ specimens tested positive by reference-standard PRNT; COVID-19 Ab−, SARS-CoV-1, hepatitis, HIV, syphilis, and preoutbreak sera specimens tested negative by the reference-standard PRNT-50.

b Congruence: percentage of results generated by the assay in agreement with the results generated by the reference PRNT. Number of results in agreement with the reference PRNT indicated in parentheses. Two-tailed McNemar tests were conducted to analyze discordance in results for COVID-19+ specimens between alternate/surrogate SARS-CoV-2 neutralization tests compared with the reference standard SARS-CoV-2 PRNT.

The conventional virus neutralization test (cVNT) conducted by BC CDC demonstrated 95% congruence (*P* < 0.0001) and almost perfect interrater agreement with the reference-standard PRNT (κ-value 0.900; Table 4). It detected 94.7% of laboratory-confirmed COVID-19 nAb+ specimens and the COVID-19 specimen that tested nAb− by the reference standard also tested negative by the cVNT. The fluorescence-activated cell sorter (FACS)-based surrogate virus neutralization test (sVNT) by CRCHUM was performed as described previously (6) and demonstrated 92.5% congruence (*P* < 0.0001) and almost perfect interrater agreement with the reference-standard PRNT (κ-value 0.851). It detected 100% of laboratory-confirmed COVID-19 nAb+ specimens. However, the COVID-19 nAb− specimen tested positive by this assay.

Luciferase-based lentivirus sVNTs performed by McGill University, CRCHUM, and Mount Sinai demonstrated 82.5% (*P* < 0.0001), 92.5.0% (*P* < 0.0001), and 85.0% (*P* < 0.0001) congruence with the reference-standard PRNT, respectively (Table 4). This corresponded with substantial, almost perfect, and substantial interrater agreement with the reference-standard PRNT (κ-value 0.655, 0.849, and 0.704, respectively). These luciferase-based lentivirus sVNTs performed by McGill University and Mount Sinai detected 100% of COVID-19 nAb+ specimens. The sVNT conducted by CRCHUM detected 89.5% of COVID-19 nAb+ specimens.

The commercially available Genscript sVNT conducted by McGill University demonstrated 95% congruence (*P* < 0.0001) and almost perfect interrater agreement (κ-value 0.900) with the reference-standard PRNT. The Genscript sVNT detected 100% of COVID-19 nAb+ specimens; the COVID-19 nAb− specimen tested positive by this assay.

Lastly, the anti-RBD IgG ELISA conducted by CRCHUM demonstrated 97.5% congruence (*P* < 0.0001) and almost perfect interrater agreement (κ-value 0.950) with the reference-standard PRNT. The ELISA detected 100% of COVID-19 nAb+ specimens; the COVID-19 nAb− specimen tested negative by this assay.

## DISCUSSION

The public health, economic, and societal impacts of the COVID-19 pandemic have led to expedited approval and emergency use of several promising vaccine candidates (https://www.who.int/news/item/15-02-2021-who-lists-two-additional-covid-19-vaccines-for-emergency-use-and-covax-roll-out). However, since much remains unknown about long-term immunity against SARS-CoV-2, reliable methods to quantify functional humoral immune responses will be indispensable for understanding protection in both vaccinated and naturally infected individuals. In this study, we validated a laboratory-developed method of quantifying antibodies capable of neutralizing SARS-CoV-2 *in vitro*. The validated PRNT was used to characterize a subset of laboratory-confirmed COVID-19 serological specimens and subsequently used as a reference standard for the evaluation of other platforms for the analysis of the SARS-CoV-2-neutralizing antibody response.

In this study, 82.1% and 54.1% of laboratory-confirmed COVID-19 specimens demonstrated 50% and 90% neutralization of SARS-CoV-2. Specimens collected from 15 to 21 days post-symptom onset demonstrated the highest average PRNT-50 titers compared with those from other time points. These observations align with previous studies that have shown variability in the development, potency, and longevity of neutralizing antibody responses in COVID-19 patients (4, 8, 21). However, specimens used in this study were not collected from the same patients at each different time point. Therefore, we are unable to determine whether the lower titers observed at later time points were due to variability in individual responses within subgroups or a temporal trend in host response.

The validated PRNT in this study demonstrated excellent intraassay and interassay precision with results falling within 2-fold of the median test result. However, the proportion of results equal to the median result between assay runs was lower when using the PRNT-90 analyses. Similarly, interassay variation has been demonstrated in PRNT assays for dengue virus, which have been used for the detection of neutralizing antibodies for decades (22, 23). Possible factors contributing to interassay variation include batch-to-batch variation in cell culture reagents, differences in cell passage number, and technician subjectivity in enumerating plaques. Future studies may wish to evaluate the difference in precision of a PRNT-75, which uses 75% neutralization of SARS-CoV-2 as a threshold for neutralization. In a previous study of dengue virus PRNT assays, PRNT-75 was identified as the optimal evaluation point with the least inter- and intraassay variance compared with that of assay thresholds from PRNT-40 to PRNT-90 (22). To date, PRNT-50 and/or PRNT-90 are the most commonly used for the evaluation of SARS-CoV-2-neutralizing antibodies.

The validated PRNT was then used as a reference standard to compare several alternate PRNTs and sVNTs for SARS-CoV-2-neutralizing antibodies. Both of the alternate PRNTs conducted by U of A and Wadsworth demonstrated excellent interrater agreement with the reference-standard PRNT. The only difference between the two assays was their result for the laboratory-confirmed COVID-19 specimen that tested nAb− by the reference standard. The specimen tested positive by an ELISA detecting anti-SARS-CoV-2 nucleocapsid IgA, IgM, and IgG antibodies (Platelia SARS-CoV-2 Total Ab assay, BIO-RAD). However, the specimen tested negative by anti-SARS-CoV-2 spike 1 ELISAs, including ZEUS ELISA SARS-CoV-2 IgG test system and Euroimmun anti-SARS-CoV-2 ELISA (IgG and IgA). While the U of A assay agreed with the reference standard, the Wadsworth assays deemed the specimen to be positive for SARS-CoV-2-neutralizing antibodies. This observation could be interpreted in 2 different ways. First, one could consider the Wadsworth assay to demonstrate sensitivity greater than that of the reference standard and U of A PRNTs for detecting nAbs in laboratory-confirmed COVID-19 specimens. This is supported by the positive results generated by the cVNT (BC CDC) and the luciferase-based sVNT (CRCHUM). The second interpretation could consider the reference standard and U of A PRNTs to have a higher threshold for neutralizing activity. This is supported by the specimen testing negative for antibodies against the SARS-CoV-2 spike 1, which contains the RBD targeted by the majority of nAbs. Further research is needed to establish the threshold of neutralizing activity *in vitro* that correlates with neutralization and protection *in vivo*. One difference was that heat inactivation of serological specimens was conducted for the reference standard and U of A assays but not for the Wadsworth assay. Heat inactivation of serum at 56°C for 30 min is standard procedure in order to inactivate complement when evaluating neutralization (24, 25). Complement has been demonstrated to enhance *in vitro* neutralization of viruses, including human cytomegalovirus (26), influenza virus (27), West Nile virus (28), and hepatitis C virus pseudotypes (29), directly and/or by enhancement of antibody neutralization. A recent study demonstrated that heat-inactivating serum specimens prior to immunoanalysis resulted in significantly lower observed levels of IgM and IgG anti-SARS-CoV-2 antibodies by ELISA (30). However, the effect was not characterized for PRNT and it remains unknown whether complement contributes to direct neutralization and/or enhancement of antibody-mediated neutralization in SARS-CoV-2 PRNT assays.

The other assays evaluated in this study demonstrate potential alternatives for the measurement of neutralizing antibodies against SARS-CoV-2. For example, aside from the alternate PRNTs, the cVNT (BC CDC) demonstrated the greatest interrater agreement with the reference-standard PRNT and offers practical benefits, such as a 96-well format to evaluate more samples per plate and decreased processing time due to the absence of a semisolid overlay step. However, like the PRNT, the cVNT still requires CL-3 facilities to manipulate live SARS-CoV-2 and analysis of neutralization can be affected by technician subjectivity. In contrast, the luciferase-based pseudotyped lentivirus sVNTs, the commercially available sVNT, and anti-RBD IgG ELISA are analyzed using automated platforms, decreasing processing time and observer subjectivity. In addition, these platforms can be conducted safely without a CL-3 facility, greatly expanding their use in lower-containment-level laboratories. However, cell-based assays, such as PRNT, cVNT, and luciferase-based pseudotyped lentivirus sVNTs, require highly skilled personnel and several days to conduct, hindering their practicality for use in large-scale immunity studies. While both anti-RBD IgG ELISAs and the commercially available sVNT are simple, high-throughput, and demonstrate excellent interrater agreement with the reference-standard PRNT, the anti-RBD IgG ELISA is a less expensive method to screen for the presence of potential neutralizing antibodies. Furthermore, the anti-RBD IgG ELISA has demonstrated near perfect agreement with another commercial sVNT (31). Thus, this ELISA could be used as a more cost-effective alternative to the commercially available sVNT as a screening assay prior to confirmation by PRNT, similar to a previously proposed algorithm (32).

It is crucial to recognize the small number of specimens in the panel used for the evaluation of these assays, which could limit the generalizability of the study. However, several of these protocols have been characterized further and demonstrated excellent performance in other studies (6, 7, 33–38). Importantly, our study demonstrates the proof-of-principle of using a sufficiently validated platform as a reference standard to directly compare nine serological methods to quantify and/or estimate the presence of neutralizing antibodies across several laboratories. Future evaluation of other alternate and surrogate platforms for the quantification of SARS-CoV-2-neutralizing antibodies will require a larger sample subset similar to the subset used for the validation of the reference-standard PRNT used in this study. Recently, the WHO has made recommendations to express serological data for neutralization in international units (IUs) as a way to effectively compare results of several studies (39). This standardization of results should be used in future studies using any of the assays evaluated in this study, especially in clinical studies, where it is crucial to be able to draw comparisons between trials.

## MATERIALS AND METHODS

### Ethics and biosafety statements.

This study was approved by the Public Health Agency of Canada’s Research Ethics Board (Protocol no. 2020-004P). All experiments involving SARS-CoV-2 (risk group 3 [RG-3]) were conducted in a containment level 3 (CL-3) laboratory. Procedures involving live SARS-CoV-2 were conducted using personal protective measures and operational practices for the manipulation of SARS-CoV-2 outlined by the Government of Canada (40).

### Cell line and virus.

Vero E6 cells (CRL-1586; ATCC, Manassas, VA), HEK 293T cells (CRL-3216; ATCC), and HEK 293T-ACE2 cells were maintained in Dulbecco’s modified Eagle’s medium, high glucose with l-glutamine (DMEM; HyClone, San Angelo, TX) supplemented with 10% heat-inactivated fetal bovine serum (FBS; Gibco, Waltham, MA). The 293T-ACE2 has been described elsewhere (6).

SARS-CoV-2 (hCoV-19/Canada/ON_ON-VIDO-01-2/2020, EPI_ISL_425177) p3 stocks were produced by infecting Vero E6 cells at a multiplicity of infection (MOI) of 0.1. After 1 h of adsorption at 37°C and 5% CO_2_, DMEM supplemented with 2% FBS was added and flasks were placed in a 37°C incubator in an atmosphere of 5% CO_2_. The flasks were monitored daily under a light microscope to detect the presence of cytopathic effect (CPE) in infected cells and lack of CPE in the mock-infected cells. After 72 h postinfection (HPI), virus supernatant was collected and centrifuged for 10 min at 525 × *g* and 4°C to remove cell debris. Aliquots of SARS-CoV-2 were prepared and transferred to a −80°C freezer for long-term storage. SARS-CoV-2 stocks were titrated using a previously described plaque assay (41).

### Samples.

Samples used for the validation of the reference standard SARS-CoV-2 PRNT were as follows: SARS-CoV-2 antibody-positive specimens comprised human serum and plasma samples from COVID-19 patients from Sunnybrook Hospital who were confirmed positive by molecular testing (Toronto Invasive Bacterial Diseases Network, ON; *n* = 218). All participants provided informed consent for collection of serum (Sinai Health Sytem REB no. 02118-U and no. 05-0016-C). The subset included 38, 60, 20, 16, 20, 22, 20, and 21 specimens collected at 1 to 7, 8 to 14, 15 to 21, 22 to 28, 29 to 35, 36 to 42, 43 to 49, and ≥50 days post-symptom onset. Of these samples, 24 were used for evaluating intraassay precision and 18 of these samples were used for evaluating interassay precision of the reference-standard PRNT. SARS-CoV-2 antibody-negative human serum samples were obtained from healthy adults prior to the COVID-19 pandemic (*n* = 52). The sample subset used for the multilaboratory comparison of alternative and surrogate platforms for the detection of SARS-CoV-2 were as follows: 19 specimens collected from laboratory-confirmed COVID-19 patients and confirmed positive for neutralizing antibody by reference standard SARS-CoV-2 PRNT (COVID-19 nAb+) and 1 specimen collected from molecularly confirmed COVID-19 patients and confirmed negative for neutralizing antibody negative by reference-standard PRNT (COVID-19 nAb−). In addition, the panel comprised laboratory-confirmed specimens from 2 SARS-CoV-1 patients, 5 hepatitis patients, 5 syphilis patients, and 3 HIV patients, as well as 5 specimens from healthy individuals collected prior to the COVID-19 pandemic. The latter were collected from individuals whose occupations required routine quantification of anti-rabies virus-neutralizing antibody titers postvaccination.

### SARS-CoV-2 plaque-reduction neutralization test.

The SARS-CoV-2 PRNT was adapted from a previously described method for SARS-CoV-1 (42). Serological specimens were diluted 1:10 in DMEM supplemented with 2% FBS and inactivated at 56°C for 30 min. In a 96-well plate, sera were further diluted 2-fold from 1:10 to 1:640 in DMEM supplemented with 2% FBS in a volume of 150 μL. One hundred fifty microliters of SARS-CoV-2 diluted at 100 PFU/100 μL was added to each well, yielding final serum dilutions of 1:20 to 1:1,280 and final virus concentrations of 50 PFU/100 μL. No neutralization, 50% neutralization, and 90% neutralization controls were prepared by diluting SARS-CoV-2 at 50 PFU/100 μL, 25 PFU/100 μL, and 5 PFU/100 μL, respectively. These were incubated with no sera. DMEM supplemented with 2% FBS was used as a no-virus control. After 1 h of incubation at 37°C and 5% CO_2_, 100 μL of each sera-virus mixture containing 50 PFU of SARS-CoV-2 was added in duplicate to 12-well plates containing Vero E6 cells at 95 to 100% confluence. One hundred microliters of each control was added in triplicate to two sets of 12-well plates containing Vero E6 cells. All plates were incubated at 37°C and 5% CO_2_ for 1 h. After adsorption, a liquid overlay was prepared by adding equal volumes of 3% carboxymethylcellulose (CMC) and 2× modified Eagle’s medium (Temin’s modification), no phenol red (2× MEM; Gibco, Waltham, MA) supplemented with 8% FBS, 4 mM l-glutamine, 2× nonessential amino acids, and 1.5% sodium bicarbonate. A total of 1.5 mL of liquid overlay was added to each well and plates were incubated at 37°C and 5% CO_2_ for 3 days. The liquid overlay was removed and cells were fixed with 10% neutral buffered formalin for 1 h at room temperature. The monolayer in each well was stained with 100 μL 0.5% crystal violet (wt/vol) in 20% ethanol for 10 min and washed with 20% ethanol. For each specimen, the average number of plaques was calculated for each dilution and compared with the average number of plaques for 50% neutralization and 90% neutralization controls. The reciprocal of the highest serum dilution resulting in 50% and 90% reduction in plaques compared with controls was defined as the PRNT-50 and PRNT-90 endpoint titer, respectively. PRNT-50 titers and PRNT-90 titers of ≥20 were considered positive for SARS-CoV-2-neutralizing antibodies, whereas titers of <20 were considered negative for SARS-CoV-2-neutralizing antibodies. Under the assumption that these specimens are negative for neutralizing antibodies, these specimens have a value of 0 for statistical analysis purposes.

### Validation of reference standard SARS-CoV-2 PRNT.

Following guidelines outlined by the World Health Organization (https://www.who.int/medicines/areas/quality_safety/quality_assurance/28092018Guideline_Validation_AnalyticalMethodValidation-Appendix4_QAS16-671.pdf), the precision, accuracy, and specificity of the SARS-CoV-2 PRNT were assessed using both PRNT-50 and PRNT-90 titers. The intraassay precision of the PRNT was defined as the agreement of results generated by a single assay for the same homogenous sample. Intraassay precision was assessed by testing samples collected from laboratory-confirmed SARS-CoV-2 patients (*n* = 24) in triplicate in a single assay run. The interassay precision of the PRNT was defined as the agreement of results generated by several assays for the same homogenous sample. Interassay precision was assessed by testing samples collected from laboratory-confirmed SARS-CoV-2 patients (*n* = 18) in three independent assay runs by three different analysts. Intraassay and interassay precision were considered acceptable if ≥90% of the observed results were within 4-fold difference of the median titer for ≥80% of positive samples tested. For both intra- and interassay validations, the sample subset comprised high-volume specimens representing a variety of PRNT titers. Samples collected from laboratory-confirmed SARS-CoV-2 patients were used even if negative for SARS-CoV-2-neutralizing antibody; this was to ensure that even negative results were congruent within and between assay runs. Accuracy of the PRNT was defined as the degree of agreement of test results with the true value. Accuracy was assessed by testing laboratory-confirmed SARS-CoV-2 undiluted and diluted at 1:4, 1:16, and 1:64 in PBS 5 times each within the same assay run. The expected titer for each diluted specimen was defined as the mean of the four observed titers for the corresponding undiluted specimen divided by the dilution factor (4, 16, and 64, respectively). Accuracy was considered acceptable if the absolute difference of the log_2_ observed mean titer and the log_2_ expected titer was ≤2.00 for ≥80% results for each dilution. Specificity of the PRNT was defined as the percentage of SARS-CoV-2 antibody-negative samples from healthy individuals collected prior to the COVID-19 pandemic, which tested negative in the assay. A specificity of ≥95% was considered acceptable.

### Alternate PRNTs.

Alternate PRNTs were conducted by Wadsworth Centre (New York, NY, USA) and the University of Alberta (Edmonton, AB, Canada; REB Pro00099761). These assays were conducted similarly to the reference-standard PRNT, with a few distinct differences in SARS-CoV-2 stock production, virus dilution, challenge and adsorption, overlay, and visualization. These differences are summarized in Table 5.

**TABLE 5:** Differences between the reference-standard PRNT and alternate PRNTs

Characteristic	Reference-standard PRNT (NML)	Alternate PRNT (Wadsworth)	Alternate PRNT (University of Alberta)
SARS-CoV-2 stock production	P4, Vero E6, DMEM, 2% FBS	P4, Vero E6, MEM, 2% FBS	P4, Vero cells, improved minimum essential medium (IMEM), 2% FBS
Specimen inactivation	56°C, 30 min	56°C, 30 min	56°C, 30 min
Specimen dilution before challenge	2-fold in DMEM, 2% FBS (1:10 to 1:320)	2-fold in MEM, 2% FBS (1:10 to 1:320)	2-fold in IMEM, 2% FBS (1:10 to 1:320)
Virus dilution	100 PFU/100 μL	200 PFU/100 μL	100 PFU/100 μL
Neutralization	96-well plate, 1:1 sera-virus mixture, 37°C, 1 hr	96-well plate, 1:1 sera-virus mixture, 37°C, 1 hr	96-well plate, 1:1 sera-virus mixture, 37°C, 1 hr
Challenge and adsorption	12-well plate, Vero E6, sera-virus mixture containing 50 PFU/well, 37°C, 1 hr	6-well plate, Vero E6, sera-virus mixture containing 100 PFU/well, 37°C, 1 hr	12-well plate, Vero, sera-virus mixture containing 50 PFU/well, 37°C, 1 hr
Overlay	1.5% carboxymethylcellulose (CMC) in MEM (no phenol red), 4% FBS	0.6% oxoid agar in MEM, 4% FBS	1% CMC in MEM, 0% FBS
Incubation time	37°C, 72 hr	37°C, 48 hr	37°C, 72 hr
Visualization	Fixation: 10% formalin, room temp, 1 hour; staining: 0.5% crystal violet in 20% ethanol; room temp, 10 min	Second overlay: 0.6% oxoid agar in MEM, 4% FBS, 0.5% neutral red, 37°C, 24 hr	Fixation-staining: 1.3% crystal violet in 11.1% formaldehyde, room temp, 1 hr
Output and interpretation	PRNT_50_ = reciprocal of the highest serum dilution resulting in 50% and 90% reduction in plaques compared with controls; positive: PRNT_50_ ≥ 20; negative: PRNT_50_ < 20	PRNT_50_ = reciprocal of the highest serum dilution resulting in 50% and 90% reduction in plaques compared with controls; positive: PRNT_50_ ≥ 20; negative: PRNT_50_ < 20	PRNT_50_ = reciprocal of the highest serum dilution resulting in 50% and 90% reduction in plaques compared with controls; positive: PRNT_50_ ≥ 20; negative: PRNT_50_ < 20

### SARS-CoV-2 microneutralization test: cVNT.

A cell culture-based SARS-CoV-2 microneutralization test was developed and conducted in a CL-3 facility at the British Columbia Centre for Disease Control (BC CDC). Sera were incubated at 56°C for 30 min to inactivate the complement. Each serum was subjected in duplicate to 2-fold serial dilution from 1:8 to 1:4,096 in volumes of 100 microliters in a 96-well microtiter plate. To each dilution, 100 50% tissue culture infective dose (TCID_50_) units of SARS-CoV-2 were added in a 50-μL volume and the preparations were incubated at 37°C for 2 h. Volumes of 100 μL of each dilution were inoculated into respective wells of a microtiter plate containing monolayers of Vero-E6 cells in MEM containing 2% fetal bovine serum. The cultures were incubated at 37°C in a CO_2_ incubator and examined after 72 h for the development of characteristic cytopathic effect. The reciprocal dilution below the one at which cytopathic effect could be detected was deemed the titer of neutralizing antibody. Titers of ≥8 were considered positive for SARS-CoV-2-neutralizing antibodies, whereas titers of <8 were considered negative for SARS-CoV-2-neutralizing antibodies. For each assay, previously tested sera were used as positive and negative controls.

### Luciferase-based lentiviral sVNTs.

Luciferase-based sVNTs using recombinant lentiviruses expressing SARS-CoV-2 spike were conducted by McGill University (Montreal, QC, Canada), Centre de recherche du CHUM (Montreal, QC, Canada), and Mount Sinai Hospital (Toronto, ON, Canada). Differences between these assays are summarized in Table 6.

**TABLE 6:** Differences between the luciferase-based lentiviral sVNTs

Characteristic	Description for:		
	Luciferase-based lentiviral sVNTs (McGill University)	Luciferase-based lentiviral sVNTs (CRCHUM)	Luciferase-based lentiviral sVNT (Mount Sinai Hospital)
Plasmids	(1) pSPAX2 (Addgene, 12260), (2) Luc (Addgene,17477), (3) SARS-CoV-2 S (Institute of Medicinal Biotechnology, Chinese Academy of Medical Sciences)	(1) pNL4.3 R-E-Luc, (2) SARS-CoV-2 S plasmid	(1) psPAX2 (Addgene, 12260), (2) Luc2-IRES-ZsGreen (BEI, NR52516), (3) SPIKE(fixK) (BEI, NR-52514)
Plasmid ratio	1:2:1	5:4	3.25:3.25:1
Production of pseudotyped lentivirus	Polyethylenimine, HEK293T, DMEM, 5% FBS, 37°C, 40 HPT	Calcium phosphate method, HEK293T, DMEM, 5% FBS, 37°C, 48 HPT	JetPrime transfection system, HEK293T, DMEM, 10% FBS, 37°C, 8 HPT, replace: DMEM 5% FBS, 37°C, 16 hr, transfer: 33°C, 24 hr
Target cell preparation	HEK293T-ACE2 (created in-house), DMEM, 5% FBS, 1 × 10^4^ cells/well, 96-well, 37°C, day before running assay	HEK293T-ACE2 (created in-house [6]), DMEM, 5% FBS, 1 × 10^4^ cells/well, 96-well, 37°C, day before running assay	HEK293T-ACE2/TMPRSS2, H10 (created in-house [34]), 10% FBS, 1 × 10^4^ cells/well, 96-well, 37°C, 1 day before running assay
Specimen dilution after pseudovirus addition	5-fold in DMEM, 2% FBS, 1:2.5 to 1:1,562.5	5-fold in DMEM, 5% FBS, 1:50 to 1:31,250	2.5-fold in DMEM, 5% FBS, 1:32 to 1:32,000
Neutralization	1:1 serum-virus mixture, 37°C, 1 hr	1:1 serum-virus mixture, 37°C, 1 hr	1:1 serum-virus mixture, 37°C, 1 hr
Challenge and incubation	37C, 2 hr, remove inoculum, replace with DMEM 10% FBS, 37°C 48 hr	37°C, 48 hr	37°C, 48 hr
Cell lysis	Steady-Glo luciferase assay system (Promega)	30 μL passive lysis buffer (Promega), 1 freeze-thaw cycle	Bright-Glo luciferase reagent (Promega)
Substrate	Steady-Glo luciferase assay system (Promega)	100 μL luciferin buffer, 50 μL 1 mM d-luciferin potassium salt	Bright-Glo luciferase reagent (Promega)
Output and interpretation	ID_50_ = plasma dilution inhibiting 50% of infection of 293T-ACE2; negative: ID_50_ < 1:2.5; positive: ID_50_ ≥ 1:2.5	ID_50_ = plasma dilution inhibiting 50% of infection of 293T-ACE2; negative: ID_50_ < 1:50; positive: ID_50_ ≥ 1:50	ID_50_ = plasma dilution inhibiting 50% of infection of 293T-ACE2; negative: ID_50_ < 10^−1.5^; negative: ID_50_ ≥ 10^−1.5^

**McGill University.** The SARS-CoV-2 spike (S) protein pseudotyped lenti-Luc virus was produced by transfecting HEK293T cells in a 10-cm dish with 3 μg of pSPAX2 (Addgene, 12260), 6 μg of Lent-Luc (Addgene,17477), and 3 μg of SARS-CoV-2 S (provided by Shan Cen, Institute of Medicinal Biotechnology, Chinese Academy of Medical Sciences) plasmid DNA using PEI (polyethylenimine). Forty hours posttransfection (HPT), viruses in the culture supernatants were collected, clarified by centrifugation at 3,000 rpm (Beckman GR-6S centrifuge) at 4°C to remove cell debris, and aliquoted, stored at −80°C. The quantity of virus stock was determined by measuring the copy number of viral RNA with reverse transcriptase-quantitative PCR (FRT-qPCR). The infectivity of the virus stock was measured by infecting HEK293T cells engineered to express ACE2 protein. The serological samples were first inactivated at 56°C for 30 min, then diluted in 100 μl DMEM containing 2% FBS in 5-fold from 1:2.5 to 1:1,562.5, mixed with 100 μl of lenti-Luc-SARS-CoV-2-S pseudotyped virus (8 × 10^4^ copies of viral RNA, diluted in DMEM with 2% FBS). After incubation for 1 h at 37°C and 5% CO_2_, virus/serum mixture was added to HEK293T-ACE2 cells that were seeded in a 96-well plate 1 day prior to infection (18,000 cells/well). Each serum dilution was tested in three independent infections. After 2 h infection, virus/serum inoculum was replaced with 100 μl DMEM containing 10% FBS. Cells were cultured for 48 h at 37°C and 5% CO_2_, and then 100 μl of 2× luciferase substrate (Steady-Glo luciferase assay system, Promega) was added for 20 min to lyse cells. Luciferase activity was measured with PerkinElmer EnSpire multimode plate reader. Luciferase values of infection without exposure to patient serum serve as the control infection. Fifty percent plaque-reduction/neutralization titer (PRNT_50_) values were calculated using the GraphPad Prism software. A sample with a half-maximal inhibitory dilution (ID_50_) of <1:2.5 was considered “negative” and ≥1:2.5 was considered “positive” for SARS-CoV-2-neutralizing antibodies.

**CRCHUM.** The luciferase-based lentiviral sVNT was conducted as described previously (6). Single-round lentivirus particles expressing SARS-CoV-2 spike and luciferase were rescued by calcium phosphate transfection of 293T cells with the lentiviral vector pNL4.3 R-E-Luc (NIH AIDS Reagent Program) and a plasmid encoding SARS-CoV-2 spike (strain Wuhan-Hu-1, GenBank accession number MN908947.3) kindly provided by Stefan Pöhlmann (17) at a ratio of 5:4. Cell supernatants were harvested 48 HPT and stored at −80°C until use. Recombinant 293T cells expressing ACE2 (HEK293T-ACE2) were seeded at a density of 1 × 10^4^ cells/well in 96-well luminometer-compatible tissue culture plates (Perkin Elmer) 24 h before infection. Recombinant virus in a final volume of 100 μL was incubated with serological specimens diluted 5-fold from 1:50 to 1:31,250 for 1 h at 37°C. The mixtures were then added to the target cells and incubate at 37°C for 48 h. Cells were lysed by the addition of 30 μL of passive lysis buffer (Promega) followed by one freeze-thaw cycle. An LB941 TriStar luminometer (Berthold Technologies) was used to measure the luciferase activity of each well after the addition of 100 μL of luciferin buffer (15 mM MgSO_4_, 15 mM KPO_4_ [pH 7.8], 1 mM ATP, and 1 mM dithiothreitol) and 50 μL of 1 mM d-luciferin potassium salt (Prolume). The neutralization half-maximal inhibitory dilution (ID_50_) was determined using a normalized nonlinear regression and represents the plasma dilution to inhibit 50% of the infection of 293T-ACE2 cells by recombinant virus. A sample with an ID_50_ of <1:50 was considered negative and ≥1:50 was considered positive for SARS-CoV-2-neutralizing antibodies.

**Mount Sinai.** The luciferase-based lentiviral sVNT was conducted as described previously (34, 35). Lentivirus particles were rescued from HEK293T cells in a 6-well plate containing 2 mL growth medium (10% FBS, 1% penicillin/streptomycin [pen/strep] in DMEM). Cells were transiently cotransfected with 1.3 μg psPAX2 (Addgene, 12260), 1.3 μg pHAGE-CMV-Luc2-IRES-ZsGreen-W (BEI, NR-52516), and 0.4 μg HDM-IDTSpike-fixK (BEI, NR-52514) using 8 μL JetPrime (Polyplus-transfection SA, 114–01) in 500 μL JetPrime buffer. After 8 HPT, the medium was replaced by 3 mL of DMEM containing 5% heat-inactivated FBS and 1% pen/strep, and the cells were incubated for 16 h at 37°C and 5% CO_2_. Cells were then transferred to 33°C and 5% CO_2_ for an additional 24 h. At 48 HPT, the supernatant was collected, centrifuged at 500 × *g* for 5 min at room temperature, filtered through a 0.45-μm filter, and frozen at −80°C until use. For the neutralization assay, 2.5-fold serial dilutions of the serum samples were incubated with diluted virus at a 1:1 ratio for 1 h at 37°C before being transferred to plated HEK293-ACE2/TMPRSS2 cells and incubated for an additional 48 h at 37°C and 5% CO_2_. After 48 h, cells were lysed, and Bright-Glo luciferase reagent (Promega, E2620) was added for 2 min before reading with a PerkinElmer Envision instrument. A sample with an ID_50_ of <10^−1.5^ was considered negative and ≥10^−1.5^ was considered positive for SARS-CoV-2-neutralizing antibodies.

### Commercially available hACE2 receptor-blocking antibody assay.

The commercially available SARS-CoV-2 sVNT (L00847; GenScript, Piscataway, USA) was performed according to the manufacturer’s instructions at McGill University (Montreal, QC, Canada). Briefly, serological specimens were incubated with horseradish peroxidase-conjugated RBD (HRP-RBD) at 37°C for 30 min. The mixtures were added to the hACE2-coated capture plate and incubated at 37°C for 15 min. Plates were then washed, removing HRP-RBD-neutralizing antibody complexes and allowing unbound HRP-RBD and HRP-RBD-nonneutralizing antibody complexes to remain bound to hACE2. 3,3′,5,5′-Tetramethylbenzidine (TMB) solution was added and allowed to incubate at room temperature for 15 min, and the reaction was stopped by stop solution. The optical density (OD) of each well was measured by spectrophotometry at 450 nm. The percent inhibition of a sample was calculated as (1 − average OD of sample/average OD of negative control) × 100%. A sample with a percent inhibition of <20% was considered negative and ≥20% was considered positive for SARS-CoV-2-neutralizing antibodies.

### Flow cytometry-based detection of antibodies against SARS-CoV-2 spike.

The flow cytometry-based assay evaluated in this study was developed and conducted by CRCHUM as described previously (43). Briefly, HEK293T cells transfected with an expression plasmid for the SARS-CoV-2 spike were stained with serological specimens diluted at 1:250. Transfected cells were stained with anti-RBD-Cr3002 monoclonal antibody diluted at 5 μg/mL as a positive control. Alexa Fluor-647-conjugated goat anti-human IgG (H+L) (Invitrogen, Rockford, IL) was used as a secondary antibody to detect IgG bound to SARS-CoV-2 spike. An LSRII cytometer (BD Biosciences, Mississauga, ON, Canada) was used to acquire the specimens. FlowJo v10.5.3 (Tree Star, Ashland, OR) was used for data analysis. A specimen was considered positive for nAbs if the proportion of cells bound by anti-spike antibodies was greater than the seropositivity threshold, which was calculated as the mean median fluorescence intensity (MFI) of all COVID-19-negative plasma plus 3 standard deviations of the mean MFI of all COVID-19-negative plasma plus interassay coefficient of variability.

### Anti-RBD IgG ELISA.

The SARS-CoV-2 RBD ELISA was used as described previously (6, 7). Briefly, recombinant SARS-CoV-2 S RBD protein (2.5 μg/mL), or bovine serum albumin (BSA) (2.5 μg/mL) as a negative control, was prepared in phosphate-buffered saline (PBS) and adsorbed to plates (MaxiSorp Nunc) overnight at 4°C. Coated wells were subsequently blocked with blocking buffer (Tris-buffered saline [TBS] containing 0.1% Tween 20 and 2% BSA) for 1 h at room temperature. Wells were then washed four times with washing buffer (TBS containing 0.1% Tween 20). CR3022 monoclonal antibody (MAb) (50 ng/mL) or a 1:250 dilution of plasma samples was prepared in a diluted solution of blocking buffer (0.1% BSA) and incubated with the RBD-coated wells for 90 min at room temperature. Plates were washed four times with washing buffer followed by incubation with secondary antibodies (Abs; diluted in a diluted solution of blocking buffer [0.4% BSA]) for 1 h at room temperature, followed by four washes. HRP enzyme activity was determined after the addition of a 1:1 mix of Western Lightning oxidizing and luminol reagents (Perkin Elmer Life Sciences). Light emission was measured with a LB942 TriStar luminometer (Berthold Technologies). Signal obtained with BSA was subtracted for each plasma and was then normalized to the signal obtained with CR3022 present in each plate.

### Statistical analyses.

A two-way analysis of variance (ANOVA) with Tukey multiple comparison posttest was conducted to analyze the relationship between reciprocal endpoint neutralizing antibody titers and time of collection post-symptom onset in the laboratory-confirmed COVID-19 patient subset. Results generated from alternative conventional and surrogate SARS-CoV-2 neutralization tests were categorized as either positive or negative for SARS-CoV-2-neutralizing antibodies as described above. Congruence was defined as the percentage of results generated by the assay in agreement with the results generated by the reference PRNT. Number of results in agreement with the reference PRNT is indicated in parentheses. Two-tailed Fisher’s exact tests were conducted to analyze the congruence between alternate/surrogate SARS-CoV-2 neutralization tests compared with the reference standard SARS-CoV-2 PRNT. Kappa values (κ-values) as a measure of interrater agreement between alternative conventional and surrogate SARS-CoV-2 neutralization tests and the reference standard SARS-CoV-2 PRNT were also calculated using this software. Assays yielding κ-values between <0 and 1.00 indicate no agreement and perfect agreement, respectively. κ-values of 0.00 to 0.20, 0.21 to 0.40, 0.41 to 0.60, 0.61 to 0.80, and 0.81 indicate slight, fair, moderate, substantial, and almost perfect agreement, respectively (44).
