# Correction for Valcourt et al., “Evaluating Humoral Immunity against SARS-CoV-2: Validation of a Plaque-Reduction Neutralization Test and a Multilaboratory Comparison of Conventional and Surrogate Neutralization Assays”

**DOI:** 10.1128/spectrum.00553-22

**Published:** 2022-06-22

**Authors:** Emelissa J. Valcourt, Kathy Manguiat, Alyssia Robinson, Yi-Chan Lin, Kento T. Abe, Samira Mubarek, Altynay Shigayev, Zoë Zhong, Roxie C. Girardin, Alan DuPuis, Anne Payne, Kathleen McDonough, Zhen Wang, Romain Gasser, Annemarie Laumaea, Mehdi Benlarbi, Jonathan Richard, Jérémie Prévost, Sai Priya Anand, Kristina Dimitrova, Clark Phillipson, David H. Evans, Allison McGeer, Anne-Claude Gingras, Chen Liang, Martin Petric, Inna Sekirov, Muhammad Morshed, Andrés Finzi, Michael Drebot, Heidi Wood

**Affiliations:** a Zoonotic Diseases and Special Pathogens, National Microbiology Laboratory, Public Health Agency of Canada, Winnipeg, Manitoba, Canada; b Max Rady College of Medicine, University of Manitoba, Winnipeg, Manitoba, Canada; c Department of Medical Microbiology and Immunology, University of Alberta, Edmonton, Alberta, Canada; d Department of Microbiology, Mount Sinai Hospital, Sinai Health System, Toronto, Ontario, Canada; e Department of Molecular Genetics, University of Toronto, Toronto, Ontario, Canada; f Department of Laboratory Medicine and Molecular Diagnostics, Division of Microbiology, Sunnybrook Health Sciences Centre, Toronto, Ontario, Canada; g Institute of Health Policy, Management, and Evaluation, University of Toronto, Toronto, Ontario, Canada; h Faculty of Medicine, University of Toronto, Toronto, Ontario, Canada; i Wadsworth Center, New York State Department of Health, Albany, New York, USA; j Department of Medicine, McGill University, Montreal, Quebec, Canada; k British Columbia Centre for Disease Control Public Health Laboratory, Vancouver, British Columbia, Canada; l Département de Microbiologie, Infectiologie et Immunologie, Université de Montréal, Montreal, Quebec, Canada; m Centre de Recherche du CHUM, Montreal, Quebec, Canada; n Department of Medical Microbiology, University of Manitoba, Winnipeg, Manitoba, Canada

## AUTHOR CORRECTION

Volume 9, no. 3, e00886-21, 2021, https://doi.org/10.1128/Spectrum.00886-21. Page 1: This article was published on 17 November 2021 with David H. Evans missing from the byline. The byline was updated in the version posted on 11 February 2022.

